# Depressive-like behavioral response of adult male rhesus monkeys during routine animal husbandry procedure

**DOI:** 10.3389/fnbeh.2014.00309

**Published:** 2014-09-09

**Authors:** Michael B. Hennessy, Brenda McCowan, Jing Jiang, John P. Capitanio

**Affiliations:** ^1^Department of Psychology, Wright State UniversityDayton, OH, USA; ^2^California National Primate Research Center, University of CaliforniaDavis, CA, USA; ^3^Department of Population Health and Reproduction, School of Veterinary Medicine, University of CaliforniaDavis, CA, USA; ^4^Department of Psychology, University of CaliforniaDavis, CA, USA

**Keywords:** social isolation, depression, sickness behavior, cortisol, nonhuman primate, rhesus monkey

## Abstract

Social isolation is a major risk factor for the development of depressive illness; yet, no practical nonhuman primate model is available for studying processes involved in this effect. In a first study, we noted that adult male rhesus monkeys housed individually indoors occasionally exhibited a hunched, depressive-like posture. Therefore, Study 2 investigated the occurrence of a hunched posture by adult males brought from outdoor social groups to indoor individual housing. We also scored two other behaviors—lying on the substrate and day time sleeping—that convey an impression of depression. During the first week of observation following individual housing, 18 of 26 adult males exhibited the hunched posture and 21 of 26 displayed at least one depressive-like behavior. Over 2 weeks, 23 of 26 males showed depressive-like behavior during a total of only 20 min observation. Further, the behavior during the first week was positively related to the level of initial response to a maternal separation procedure experienced in infancy. In Study 3, more than half of 23 adult males of a new sample displayed depressive-like behavior during 10 min of observation each of Weeks 7–14 of individual housing. The surprisingly high frequency of depressive-like behavior in Studies 2 and 3 may have been due to recording behavior via camera with no human in the room to elicit competing responses. These results suggest that a common animal husbandry procedure might provide a practical means for examining effects of social isolation on depression-related endpoints in a nonhuman primate. The findings also suggest that trait-like differences in emotional responsiveness during separation in infancy may predict differences in responsiveness during social isolation in adulthood.

## Introduction

Social isolation and loneliness are risk factors for developing depressive illness (Barnett and Gotlib, 1988; Bruce and Hoff, 1994; Cacioppo et al., 2010) though vulnerability varies widely across individuals (Wu et al., 2013). Consequences of social isolation can be either immediate or delayed. That is, the onset of depression may either coincide with a period of social isolation, or be facilitated by experiences of separation or related stressors in early life. Indeed, differences in childhood separation experiences, or the response to those experiences, may be important contributors to the variability in vulnerability observed in adulthood (Agid et al., 1999; Belsky et al., 2007; Gillespie and Nemeroff, 2007; Heim and Binder, 2012; Ignácio et al., 2014).

Attempts to model the impact of social isolation on depression in laboratory animals date back to studies of Harlow and others examining the effects of prolonged maternal separation or isolation rearing in nonhuman primates (Kaufman and Rosenblum, 1967; Novak and Harlow, 1975; Mineka and Suomi, 1978). These approaches, now uncommon, have been largely replaced by rodent studies of behavioral and biological consequences of social isolation that may be homologous to the depressogenic effects observed in humans. For instance, social isolation in adult prairie voles increases measures of helplessness (immobility in the forced swim test; reduced struggling when suspended by the tail) and anhedonia (reduced sucrose intake) (Grippo et al., 2008; Bosch et al., 2009). In young degus (*Octogon degus*), removal from family members induces a passive, depressive-like crouched posture during isolation as well as long-term changes in a variety of neuroanatomical and neurochemical alterations in limbic and cortical regions that, in humans, may contribute to the development of depressive illness (Helmeke et al., 2008; Colonnello et al., 2011). In developing guinea pigs, a several-hour period of isolation from the mother elicits a constellation of passive responses, including a hunched or crouched posture, eye-closure, and piloerection reminiscent of the “despair” reaction of infant monkeys undergoing prolonged separation. Evidence indicates this reaction in guinea pigs is mediated by proinflammatory activity (Hennessy et al., 2009), a finding in line with the burgeoning evidence that inflammatory processes are important mediators of depressive illness in humans (Dantzer et al., 2011).

Despite the progress with rodent paradigms, a better understanding of the relevance of the rodent findings for humans would be achieved if hypotheses generated in these studies could be tested in a primate model. Ideally, such a model would be more practical and involve less extreme procedures than isolation rearing or prolonged separation of mothers and infants. One approach has been to remove adult monkeys from social groups. Limited early work indicated that separation of adult male rhesus monkeys (*Macaca mulatta*) from a family group for 10 or more weeks of individual housing induced a depressive-like reaction (Suomi et al., 1975). The subjects exhibited self-clasping with the body bent forward in the fashion of separated infants. A case report also noted a depressive-like response in a female pig-tailed macaque (*M. nemestrina*) following the death of her infant, a miscarriage, and separation from a close social companion (Rasmussen and Reite, 1982). Recent studies with adult macaques have obtained similar results. Perera et al. (2011) found adult male bonnet macaques (*M. radiata)* displayed a depressive-like reaction of a slumped body posture accompanied by an apparent lack of interest in their surroundings following 13–15 week cycles of 2 days of separation from the social group interspersed with 5 days of reunion. Similarly, Li et al. (2013) observed that 90 days of social isolation of adult female cynomolgus monkeys (*M. fascicularis*) increased the incidence of sitting with head down and apparent disinterest in the environment. In a separate study, this reaction was observed in adult female *M. fascicularis* during the course of 12 months of individual housing (Shively et al., 2005). Camus et al. (2013, 2014) observed depressive-like behavior in adult male rhesus and cynomolgus monkeys that had been singly housed for at least 9 months, though animals of both species displayed substantial inter-individual variability in susceptibility. It is noteworthy that a depressive-like behavioral reaction involving a hunched body posture was also found in a portion of adult rhesus monkeys administered the proinflammatory cytokine, interferon-alpha (Felger et al., 2007), suggesting possible mediation of depressive-like responses by proinflammatory processes as observed in separated guinea pigs.

In all, these studies indicate that the influence of social isolation on depression might be studied in adult macaque monkeys so as to provide a bridge between rodent studies and those of depressed humans. Yet, the paradigms described still require protracted separation procedures, and remain costly in terms of time and resources. Here we report observations suggesting that the depressive-like behavioral reaction in adult rhesus monkeys occurs more routinely than generally is appreciated, that it can rapidly be elicited during isolation, and that it can persist for weeks. We also explore how vulnerability to the effects of social isolation in adulthood is related to measures of responsiveness to social separation in infancy.

## General methods

### Animals and housing

The work was conducted at the California National Primate Research Center (CNPRC) at Davis, California, which maintains a colony of more than 5000 rhesus macaques, with most routinely housed in half-acre, outdoor field cages in mixed age and sex social groups of up to 160 individuals per cage. The focus of the present work was on the responses of rhesus brought from these outdoor social groups to individual housing indoors. When housed indoors, animals were maintained in standard size cages for laboratory monkeys depending on weight (0.6 m^2^ floor area for animals up to 10 kg, 0.7 m^2^ for animals up to 15 kg), with twice daily feeding, and a 12:12 light dark cycle (lights on at 0600). Animals were housed in upper and lower rows on each side of the room; animals had visual access at all times to animals on the other side of the room. All procedures were conducted according to the Guidelines for Use and Care of Laboratory Animals of the National Research Council and according to CNPRC SOPs. The CNPRC is accredited by the Association for the Assessment and Accreditation of Laboratory Animal Care. Experimental protocols were approved prior to implementation by the University of California, Davis IACUC.

### Biobehavioral assessment

Most infants born at the CNPRC undergo a biobehavioral assessment (BBA) between 3 and 4 month of age that is aimed at characterizing the behavioral and physiological responsiveness of young infants. The BBA is a highly standardized test battery assessing a variety of measures over a 25-h period [see Golub et al. (2009) for a detailed description]. As part of the procedure, all infants are observed for 5 min at the beginning and near the end of the 25-h period during which they are separated from their mothers for testing. A total of 32 activity states (e.g., sit, stand) and events (e.g., lipsmack, self-clasp) are monitored. Exploratory and confirmatory factor analysis of these scores from nearly 1500 infants yielded a two factor solution: Emotionality and Activity (Golub et al., 2009). In Studies 2 and 3 of the current report, we examine the relation of the Emotionality and Activity factor scores for Days 1 and 2 of the BBA to the depressive-like response of these animals during social isolation in adulthood.

## Study 1: estimates of prevalence

When monkeys residing in outdoor field cages at the CNPRC are assigned to an experiment, it is routine procedure for the animals to be captured and placed in an indoor colony room individually or in pairs for a period of acclimation prior to experimental manipulation. The animals often remain housed under these conditions during the course of the experiment. While indoors, the monkeys are regularly observed by behavioral management personnel to monitor welfare and address behavioral issues. Study 1 was initially prompted by reports of behavioral management staff that monkeys housed individually indoors occasionally exhibited a hunched body posture suggesting a depressive-like reaction. Animals in outdoor field cages rarely display the hunched posture, and when they do, it is taken as a sign of potential physical illness, which is frequently confirmed upon veterinary examination. On a yearly basis, hunched posture unrelated to illness is observed on average in less than 0.5% of the opportunistic biweekly scans by behavioral management staff of all animals maintained in field cages. Our first step then was to document the approximate prevalence of this reaction among the population of monkeys housed indoors.

### Methods

#### Animals and housing conditions

The population observed consisted of all rhesus monkeys housed in all indoor colony rooms at the CNPRC. The population included both males and females ranging in age from infants to full adults and maintained in rooms containing from 2 to 80 monkeys. Most animals had been transferred from outdoor housing.

#### Behavioral observations

Five-minute focal observations of all monkeys housed indoors were made monthly at variable times throughout the day as part of regular monitoring by behavioral management staff. These observations were made by observers in full view of the monkey observed. Behavioral management staff are trained to achieve excellent inter-observer reliability [Krippendorf *α* coefficient = 1; *p* (not reaching *α*-min 0.90) = 0.0000] (Hayes and Krippendorff, 2007). Here we assessed the frequency with which animals were observed engaging in a hunched posture as reflected by the “withdrawn” item from the behavioral management ethogram (see Table 1 for definition). To do so, we compiled the observations of behavior management staff over calendar year 2009.

**Table 1:** **Definitions of behavior scored for adult rhesus males in the three studies**.

*Study 1*	
Withdrawn	Sitting in hunched position with head below shoulders and eyes open for at least 30 s while not engaging in any behaviors (F)
*Studies 2 and 3*	
Hunched posture	Sitting with head the same level or lower than the shoulders; arms and limbs huddle to the center of the body; no movement of the body or the four limbs; eyes open or unable to determine whether the eyes are open or not. When huddling, the animal can yawn or scratch (D)
Lie	Relaxed posture with body resting on a horizontal surface. Weight is not supported by limbs; eyes are open (D)
Day time sleep	Sitting or lying with eyes closed (observations made during day time); if sitting, head must be above shoulders to differentiate from hunched posture (D)

*F = frequency; D = duration*.

### Results and discussion

During 2009, 129 of approximately 1800 monkeys exhibited the withdrawn behavioral category, resulting in an estimated prevalence rate of 7.2%. For individually housed rhesus (~35% of those indoors) the prevalence rate was 18.9%. For pair-housed animals, prevalence was only 0.9%. Of the 129 animals observed in this posture, 79 were adult males. We had no control over the number of monkeys housed indoors individually or in pairs at any one time. Rather animals were frequently brought from, or returned to, outdoor social groups, while others were moved back and forth from testing as dictated by individual experimental protocols. Further, individual animals were housed indoors for differing numbers of observation sessions. As a result, the prevalence rate and estimate of frequency of occurrence are only approximate. Nonetheless, the results suggest that a depressive-like response to these procedures does sometimes occur in rhesus monkeys housed indoors and that it is much more common in individually housed, than in pair-housed, animals.

## Study 2: initial response to individual housing

This study capitalized on the procedures of an unrelated experiment in which 26 adult male rhesus were brought from the outdoor field cages to indoor, individual housing under controlled, uniform conditions. The behavior of the monkeys was recorded live by use of video camera equipment located in the room; each monkey was observed on four occasions during their first 2 weeks indoors. The defining feature of the depressive-like behavior of the monkeys in Study 1 was a curled or hunched body posture. In the second study, we again examined such a posture, but we used a modified definition that more precisely specified the position of the body and criteria for the simultaneous occurrence of other behaviors. In addition, the criterion of a minimum duration of 30 s was omitted and we scored the true duration of this response. The distinctions between this category (referred to here simply as “hunched posture”) and the “withdrawn” category of Study 1 are delineated in Table 1. A hunched posture not only conveys an impression of depression, but it also is a classic “sickness behavior” that can be induced by pathogen exposure or sometimes by stress. Sickness behaviors are thought to be mediated by the same proinflammatory processes that underlie forms of depression (Dantzer et al., 2008; Anisman, 2009), in particular, perhaps, depressive episodes precipitated by stress (Miura et al., 2008; Anisman, 2009). In light of earlier findings that a superficially similar behavioral reaction during separation in guinea pigs appears mediated by proinflammatory processes (Hennessy et al., 2009) and that a response of this sort can be induced in rhesus macaques with injection of a proinflammatory cytokine (Felger et al., 2007), we also scored two other behaviors—lying down and daytime sleeping—which are unusual for healthy rhesus, particularly during active periods (e.g., mornings), but which are common sickness behaviors and also are consistent with a depressive-like behavioral reaction. Because our definitions exclude the possibility of any two of these three behaviors being scored simultaneously, we summed the time spent engaged in the three behaviors to determine a total duration of depressive-like responding. In order to assess possible signs of long-term consistency in responsiveness, we also examined the relation between the Emotionality and Activity factor scores from BBA testing during separation in infancy and the behavioral response to individual housing in adulthood.

### Methods

#### Animals and housing conditions

Twenty six adult male rhesus macaques which had undergone BBA testing in infancy, and which were currently living in outdoor field cages, were moved to individual housing at a mean age of 6.1 years (range: 5.6–7.8 years) and were housed as previously described. For transfer from field cages to individual housing, monkeys were netted by animal care staff and moved in transport cages.

#### Behavioral observations

Beginning 4 or 5 days after the relocation, behavioral observations were conducted on 12–14 animals per day 4 days per week at 0845 to 1045 h (during the period of the day when the rhesus are most active) resulting in two, 5-min observations per animal per week for 2 weeks. A stand containing two video cameras (Radio Shack VSS400), which permitted recording of behavior from monkeys in a top and bottom cage in consecutive (but randomized) order, was positioned 66 cm in front of a cage by a technician, who then moved to an adjacent room out of visual contact of the animal. Behavioral data were coded live on a monitor (Sony KV32S20) connected to the camera, using The Observer (Noldus, 1991) software (on a laptop PC) according to the predetermined random order for top vs. bottom cage. Following data collection on two animals, the technician entered the room and relocated the stand for the next pair of animals, again using a predetermined random order. The principal measures of interest (scored as duration) are defined in Table 1. Prior to any behavioral data collection, the technician established interobserver reliability of greater than 85% agreement for behaviors on the ethogram with another member of the laboratory.

### Results and discussion

Eighteen of 26 adult males exhibited the hunched posture during the first week of observations while housed individually indoors, and 14 of 26 showed this posture during the second week (Table 2). In terms of consistency, 13 of the 14 animals exhibiting the hunched posture during Week 2 had also shown the behavior the first week. Thirteen of the animals that displayed the hunched posture, as well as four that did not, exhibited lying down and/or day time sleeping across the 2 weeks. Eight monkeys showed one of the scored behaviors, 13 showed two, and two exhibited all three. At least one of these behaviors was shown by 21 of 26 (81%) animals during their first week of individual housing, 16 of 26 (62%) during the second week, and a full 23 of 26 (88%) across both weeks indoors. A Wilcoxon test showed that significantly more time was spent in depressive-like behavior during the first as compared to the second week of observation (*T* = 72, *N* = 23, *p* < 0.05). Overall, the percent of time that monkeys engaged in these behaviors varied considerably such that small subgroups appeared to be especially vulnerable and especially resilient to the isolation procedure. Although no animal evinced all three behaviors on both weeks, three monkeys exhibited the primary measure of the crouched stance plus one other depressive-like behavior on both weeks; these animals spent more than half of the observation time in depressive-like behavior. On the other hand, three monkeys showed no depressive-like behavior whatsoever in either week.

**Table 2:** **Per cent males exhibiting, and median duration of, depressive-like behaviors in Study 2**.

Behavior	First 8 days	Second 8 days
Hunched posture		
% exhibiting	69	54
Median duration (s)	57	11
Lie		
% exhibiting	19	12
Median duration (s)	0	0
Day time sleep		
% exhibiting	46	19
Median duration (s)	0	0
Total depressive-like behavior		
% exhibiting	81	62
Median duration (s)	197	37

**Figure 1:**
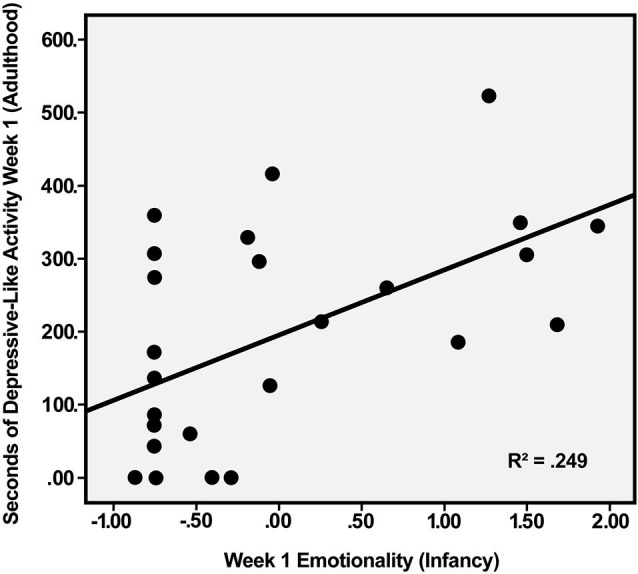
**Relation between Emotionality factor score of male rhesus monkeys during first day of social separation in infancy and duration of depressive-like behavior (maximum possible = 600 s) during first week of individual housing in adulthood, *p* < 0.02**.

Multiple regression was used to explore the relation between depressive-like behavior during the first week of indoor housing—when this behavior was most common—and the Emotionality and Activity factor scores during social separation for BBA testing in infancy. Using the predictors of Emotionality on Day 1 and Day 2 of BBA testing, analysis yielded a significant effect, *F*_(2, 23)_ = 5.15, *p* < 0.02. Of the two predictors, only Emotionality on Day 1 was significant, *R*^2^ = 0.249, *t*_(23)_ = 2.66, *p* < 0.02, indicating that the initial emotional response to separation in infancy was positively related to the initial depressive-like response when the males were moved from outdoor social groups to individual housing 5–7 years later (Figure 1). Regression analysis predicting depressive-like behavior in adulthood based on the Activity score obtained from the 2 days of the BBA approached significance, *F*_(2, 23)_ = 2.84, *p* < 0.08, with greater activity in the BBA tending to be associated with greater depressive-like responding in adulthood.

The most striking aspect of the findings of this study was the high frequency of depressive-like responding. The rate was many times greater than that observed in the first study. It would seem that if a week or two of isolation so readily evoked such a reaction in adult rhesus that this effect would have been well established in the literature by now. We suggest that a critical factor that may have obfuscated previous estimates of the frequency of the depressive-like response of adult male rhesus under these conditions is the detection of a human observer by the separated animal. In the first study, an observer was physically present in the room, whereas in the second study, all behavior was recorded by means of a camera. Anecdotally, a technician with more than 30 years of experience recording behavioral data of nonhuman primates in field and laboratory settings reported seeing little or no depressive-like reaction when entering the room in which rhesus were individually housed at the time of the second study. It may be that the presence of a human evokes an arousal response of greater motivational salience than depressive-like behavior. It is well known that proinflammatory mediated behaviors can be suppressed in the face of more pressing environmental demands (Aubert, 1999).

## Study 3: long-term responses to individual housing

Because the monkeys of Study 2 exhibited depressive-like responding throughout the first 2 weeks of isolation in which they were observed, the length of time this response persists remains unknown. To address this issue in Study 3, we examined data obtained in a fashion identical to that of Study 2 but with an additional cohort of adult males. These animals had been tested in a study that was conducted prior to Study 2; they were also from the field cages, of comparable age, and were housed in the same room as were the Study 2 animals. In this case, however, behavior observations did not begin until 6 weeks following isolation, and then continued for 8 weeks, at which time additional procedures began. In addition, we once again explored relations between responses in BBA testing and later depressive-like responding.

### Methods

#### Animals and housing condition

Twenty-four adult male rhesus macaques, who as infants had been examined in the BBA program, served as subjects. All animals were born and reared in the outdoor field cages, and were relocated to individual indoor housing cages at a mean age of 5.8 (range: 5.2–6.6) years of age. Animals were housed as described above. One animal was missing behavioral data and was dropped from analyses.

#### Behavioral observations

Beginning 6 weeks after relocation to individual housing, behavioral data were recorded for all animals between 0830 and 1030 during two, 5-min observations per animal per week; data collection occurred during 4 days each week, with half of the animals observed on each day. Data were coded live using the same equipment and procedures, and with the same inter-observer reliability, as for Study 2.

### Results and discussion

Depressive-like responding was still evident from 7 to 14 weeks following the introduction to individual housing (Table 3). Twelve of 23 animals (52%) exhibited depressive-like responding during the first week of observation and 13 of 23 (57%) did so during the 8th week. A Friedman ANOVA by ranks found no difference across weeks of observation in the duration of depressive-like behavior ($X_{r}^{2}$ = 8.40, *N* = 23, *p* > 0.20). Each animal exhibited signs of depressive-like behavior during 1 or more weeks of observation. Seven monkeys showed just one of the behaviors, 12 showed 2, and 4 displayed all 3. The sample was characterized by substantial variability across animals within each week. During 6 of the 8 weeks examined, nearly half of the monkeys (i.e., 10 or 11 of 23) showed no depressive-like responding at all, while others spent considerable time engaged in this behavior. As seen in Table 3, scores ranged up to exactly half of the 10 min (600 s) observation period on 3 separate weeks.

**Table 3:** **Per cent males exhibiting, median duration, and range of, total depressive-like behavior in Study 3**.

Week of observation								
**Depressive-like behavior**	**1**	**2**	**3**	**4**	**5**	**6**	**7**	**8**
% exhibiting	52	57	65	57	52	57	74	57
Median duration (s)	4.5	16.2	19.3	5.2	0.5	7.03	3.8	2.7
Range (s)	153.2	300.0	202.0	295.4	276.7	300.0	277.3	300.0

Especially vulnerable and resilient animals were not as obvious during the 8 weeks well into the isolation period in Study 3 as they were during the first 2 weeks of isolation in Study 1. Specifically, no monkey showed more than one of the depressive-like behaviors on each of the 8 weeks, and all animals showed depressive-like responding at some point. Nonetheless, relatively extreme groups still existed, with five monkeys displaying at least some depressive-like behavior on each of the 8 weeks, and five showing such behavior on only 1 or 2 weeks. Once again, the hunched posture was the primary component of the depressive-like behavior score. Monkeys spent a mean of 406.5 s in depressive-like behavior across the 8 weeks, of which 325.7 s (80%) were spent in the hunched posture. Twenty-one of 23 monkeys were observed in the hunched posture at some point during the 8 weeks of observation, and 18 of 23 were found to exhibit lie and/or day time sleep.

Finally, regression analysis found no significant relation between the factor scores of Emotionality and Activity during the 2 days of BBA testing and the total amount of depressive-like behavior during Weeks 7–14 of individual housing. In all, the results of Study 3 indicate that the depressive-like effects seen in adult male rhesus monkeys during the first 2 weeks of individual indoor housing are still apparent for more than 3 months.

## General discussion

The present results indicate that a standard animal husbandry procedure of removing adult male rhesus macaques from large outdoor social groups to indoor individual housing can readily elicit a depressive-like behavioral response. The general characteristics of the posture are a forward flexion of the head and kyphotic-like posture with little motor activity. Some variation of this posture, under an assortment of designations (e.g., hunched, huddled, withdrawn, slumped) has repeatedly been identified with a depressive reaction in adult and infant nonhuman as well as human primates (e.g., Spitz, 1946; Novak and Harlow, 1975; Suomi et al., 1975; Felger et al., 2007; Canales et al., 2010; Camus et al., 2013; Li et al., 2013). In one particularly well-studied example, Shively and colleagues have described the same essential posture in a subset of socially subordinate adult female cynomolgus monkeys (Shively et al., 1997; Willard and Shively, 2012). These animals also display a variety of physiological alterations, including reductions in hypothalamic-pituitary-adrenal (HPA) negative feedback, volume of the hippocampus, and 5HT_1a_ binding potential that parallel effects described in depressed human patients. Whether such changes occur in male rhesus brought to individual housing under the conditions of the present study remains to be tested.

The much higher prevalence of the withdrawn posture in singly housed than in pair-housed animals in Study one suggests that the effect was largely due to social isolation (which may engender a psychological state akin to loneliness). However, other factors such as space restriction, indoor housing, loss of mating opportunities, and stressors inherent in large populations of rhesus maintained in indoor colony rooms—such as increased noise—may also have contributed to the depressive-like response. One might argue that the hunched, singly housed animals were simply “bored” or had little else to do. It should be pointed out, though, that the animals were not housed in a stimulus-deprived environment. All monkeys received daily object (e.g., “kong” toys) and food (via foraging boards) enrichment, with fresh fruit and vegetables provided twice weekly and video enrichment weekly or biweekly. Moreover, in Studies 2 and 3, the cages in the experimental room were arranged so that each monkey could view both familiar and unfamiliar animals, with which they interacted both visually and vocally. Another possible interpretation of our results is that animals were sleeping as part of their normal daily activity budget (e.g., Post and Baulu, 1978). In this regard, it is important to emphasize that morning sleeping is very unusual in the outdoor field cages where most of the social groups are housed at the CNPRC. It has only rarely been observed during morning hours by two of the current authors (Brenda McCowan, John P. Capitanio) and their staff, with decades of experience at this facility (personal communication). Finally, interpretation of the hunched posture in terms of depression rather than normal sleep or boredom seems more parsimonious in light of its positive association with emotional responsiveness in infancy.

The depressive-like nature of the behaviors scored here is supported by their resemblance to specific symptoms of Major Depressive Disorder described in the DSM-5 (American Psychiatric Association, 2013). Specifically, the hunched posture and lying down might be regarded as indicative of either “decreased interest or pleasure” or “fatigue or loss of energy”, whereas day time sleeping corresponds to the symptom of “change in sleep”. As required by the DSM-5, the behaviors, particularly the crouched stance, were frequently observed for more than 2 weeks. While the hunched body posture suggests a depressive reaction, it also is a common “sickness behavior” induced by proinflammatory cytokines (Hart, 1988). Sickness behaviors are considered adaptive responses to pathogen exposure. However, sickness behaviors can also be elicited by stressors (Maier and Watkins, 1998), and the same proinflammatory processes that are known to induce sickness behaviors appear to be important contributors to forms of human depression as well (Dantzer et al., 2011). Thus, the possibility that inflammatory processes were mediators of the behavioral findings in the present studies may warrant further investigation.

The surprising frequency with which males exhibited the depressive-like response in Studies 2 and 3 seems likely to have been due to the recording of behavior by video camera so that no observer was visible to the subjects. The approach of a human stranger is an established laboratory stimulus to elicit defensive behavior in rhesus macaques (Willette et al., 2007; Rogers et al., 2008; Gottlieb and Capitanio, 2013). If male rhesus perceives a human observer as threatening, depressive-like responding might be suppressed in lieu of a defensive response.

The findings of the first study provide normative data on the prevalence of a depressive-like posture in a large colony of individually housed rhesus macaques over a 1-year period, at least when monitored directly by a human observer. Many experimental details differed between the first vs. the second and third studies that could have influenced the results. But to the extent that many animals in Study 1 were, in fact, suppressing depressive-like activity due to the presence of a human observer, a much greater percentage of the monkeys would be expected to have exhibited the depressive-like posture in Study 1 if observation had been by video. This raises the question of why a small percentage of animals still displayed the response despite the observer’s presence. One possibility is that these were the animals most severely affected by individual housing, i.e., that the emotional reaction to the housing conditions was of higher motivational priority than exhibiting behavior in response to the presence of the human. In any event, the results suggest that the hunched posture together with lying on the substrate and day time sleeping may be useful measures for those charged with monitoring the conditions of nonhuman primates housed individually. Indeed, our results may have significant implications for management of captive primate colonies, and lead to questions that should be examined empirically; for example, which classes of social partners might best mitigate the behavioral effects of individual, indoor housing, and can housing with a partner blunt inflammatory mechanisms that might underlie these behavioral consequences?

We observed considerable variability in the depressive-like responding of monkeys brought to individual housing. One clue as to the source of variation is provided by the BBA results. Those males showing greater initial reaction to the social separation during BBA testing at 3–4 months of age also showed greater initial depressive-like response to individual housing when 5.6–7.8 years of age. Behaviors contributing to higher Emotionality factor scores on the BBA are cooing and barking vocalizations, scratching, threat displaying, and lipsmacking. These results suggest possible trait-like differences in emotional responding identifiable at a very early age may be predictive of depressive-like reactions to social stressors in adulthood. No correlation between the Emotionality factor of the BBA and depressive-like responding during the total of Weeks 7–14 was found in Study 3. While these results could simply indicate lack of a robust association between the early and late measures, it is reasonable that the early emotional response during isolation in infancy would be most strongly related to earlier responses to isolation in adulthood. With more prolonged exposure to isolation, various individual coping strategies may come into play to mask the relation between the infant and adult responses. Prospective work examining isolation-induced responding longitudinally would help clarify this relationship.

There are limitations of our study to be acknowledged. Because we analyzed data originally collected for other purposes, some procedures were not optimal for our goals (e.g., length of behavior observation sessions; procedural differences between the first and later studies). Moreover, the general exploratory nature of our findings call for replication under *a priori* controlled conditions. Further, while the primary measure of a hunched posture is fully consistent with other studies of depressive reactions in primates and conveyed a strong impression of such a reaction here, the supplementary measures of day time sleep and lying down are open to a wider range of interpretation. Nonetheless, even with the less than optimal length of observations sessions, and even if only the primary behavioral measure of a hunched posture is considered, the results still strongly suggest that transfer from large outdoor social groups to individual indoor housing quickly induces a depressive-like reaction in a significant proportion of adult male rhesus.

In summary, the rapid emergence of a depressive-like response in adult rhesus monkeys following a standard laboratory procedure may afford an approach for examining effects of social isolation on the development of depression-related outcomes in nonhuman primates that is more practical and economical than current options. In addition, this reaction might also be a useful metric for those monitoring the welfare of captive nonhuman primates.

## Author contributions

All authors contributed to the design of individual experiments and/or the conceptual framework in which they are presented here. Michael B. Hennessy, Brenda McCowan, and John P. Capitanio analyzed the data. Michael B. Hennessy and John P. Capitanio wrote the paper. Jing Jiang collected behavioral data, and Brenda McCowan and Jing Jiang critiqued the intellectual content.

## Conflict of interest statement

The authors declare that the research was conducted in the absence of any commercial or financial relationships that could be construed as a potential conflict of interest.
